# The stress–Wnt-signaling axis: a hypothesis for attention-deficit hyperactivity disorder and therapy approaches

**DOI:** 10.1038/s41398-020-00999-9

**Published:** 2020-09-18

**Authors:** Cristine Marie Yde Ohki, Leoni Grossmann, Emma Alber, Tanushree Dwivedi, Gregor Berger, Anna Maria Werling, Susanne Walitza, Edna Grünblatt

**Affiliations:** 1 Department of Child and Adolescent Psychiatry and Psychotherapy, University Hospital of Psychiatry, University of Zurich, Zürich, Switzerland; 2 Neuroscience Center Zurich, University of Zurich and the ETH Zurich, Zürich, Switzerland; 3 Zurich Center for Integrative Human Physiology, University of Zurich, Zürich, Switzerland

**Keywords:** ADHD, Molecular neuroscience

## Abstract

Attention-deficit hyperactivity disorder (ADHD) is one of the most common psychiatric neurodevelopmental disorders in children and adolescents. Although ADHD has been studied for nearly a century, the cause and pathophysiology of ADHD is yet largely unknown. However, findings from previous studies have resulted in the formation of a new hypothesis: Apart from the well-known multifactorial etiology of ADHD, recent evidence suggests that the interaction between genetic and environmental factors and especially Wnt- and mTOR-signaling pathways might have an important role in the pathophysiology of ADHD. The Wnt-signaling pathway is known to orchestrate cellular proliferation, polarity, and differentiation, and the mTOR pathway is involved in several significant processes of neurodevelopment and synaptic plasticity. As a result, dysregulations of these pathways in a time-dependent manner could lead to neurodevelopmental delays, resulting in ADHD phenotype. This review presents further evidence supporting our hypothesis by combining results from studies on ADHD and Wnt- or mTOR-signaling and the influence of genetics, methylphenidate treatment, Omega-3 supplementation, and stress.

## Introduction

Attention-deficit hyperactivity disorder (ADHD) is a neurodevelopmental disorder characterized by inattentiveness, hyperactivity, and impulsivity^1^. It is one of the most common psychiatric and behavioral disorders in children and adolescents with over 5% of the population affected worldwide and persists in about 60% of the cases into adulthood^2^. ADHD is often accompanied by comorbidities such as conduct or oppositional defiant disorder, major depressive disorder (MDD)^3^, obsessive-compulsive disorder (OCD)^4^, personality disorder^5^, autism spectrum disorder (ASD)^6^, and epilepsy^7^ among others. Further, ADHD has also been associated with an increased risk of obesity/overweight^8^. The burden that ADHD patients and their families face in daily life greatly affects their adult life. Moreover, according to a recent review, the global burden of developmental disorders including ADHD has not significantly improved since 1990^9^.

Emotional dysregulation has been found also to be very common in ADHD. It has been suggested to be a significant cause of impairment in the different aspects of ADHD patients’ life^10,11^. ADHD has also been associated with polysubstance-dependency, early onset of addiction, and borderline personality disorder^12^. A substantial amount of negative impact on school performance and long-term academic outcomes has been found in patients with ADHD. Yet, adequate treatment is able to improve academic performances^13,14^. In addition, studies show that young people with ADHD were at a considerably higher risk of being involved in accidents^15^. ADHD diagnosis, in particular the combined type (hyperactive-impulsive subtype), was found to have an impact on reduced estimated life expectancy^16^. In addition, in adult ADHD, the assessed health-related quality of life, work productivity, and regular daily activities have been found to be impaired^17^.

Alterations in brain structure, connectivity, and maturation processes in patients with ADHD have been reported^18–22^. Particularly, a delay in cerebral cortex maturation predominantly in the frontal cortex has been noted in ADHD patients^18^. In a study combining magnetic resonance imaging (MRI), diffusion tensor imaging (DTI), and resting-state functional MRI (fMRI) technique, alterations were found in the forceps minor, the internal capsule, the corona radiata, the splenium of the corpus callosum, and the bilateral basal ganglia of patients with ADHD^23^. DTI application revealed that in adults with a history of ADHD, maturation of white matter was delayed^24^. In a recent mega-analysis by the ENIGMA-ADHD consortium, subtle differences in cortical surface area were found in children, confirming the involvement of the frontal cortex in ADHD as well^19^.

The heritability of ADHD is estimated between 77 and 88%^25^, however, no single gene alone may predict the disorder. On the contrary, several studies have indicated that a third of the heritability in ADHD is caused by the polygenic component that comprises several common variants each with small effect size^26^. Furthermore, a great deal of evidence supports the interaction between genetic (polygenic) and environmental factors during early development, in particular prenatal stress, leading to the neurobiological susceptibility to the disorder^27,28^.

Regarding pharmacological treatment, first-line agents for ADHD are stimulants such as methylphenidate (MPH) or dexamphetamine^29^. Structural and functional neuroimaging analysis of ADHD brains suggests that psychostimulants (e.g., MPH) are associated with normalizing the aforementioned structural abnormalities seen in ADHD patients^30,31^.

Although a wide range of studies has been performed to understand the underlying pathology of ADHD, the etiopathology of ADHD is still not fully understood. Elucidating the etiopathology of ADHD will help to expand the strategies to reduce symptoms, develop personalized therapy and predict response to medication therapy. New insights will additionally reveal potential biomarkers for improved differential and predictive diagnosis approaches^32^. The findings from previous studies compelled us into forming the following hypothesis: Apart from the already known multifactorial etiology of ADHD, recent evidence suggests that the interaction between genetic and environmental factors and especially Wnt- and mTOR-signaling pathways might have an important role in the pathophysiology of ADHD. In this narrative review of the literature, we will present the current evidence supporting this hypothesis linking genetic, stress, and Wnt/mTOR pathways with ADHD, as well as discuss the missing link that needs to be investigated.

### Biological pathways and their link with ADHD

#### The Wnt/mTOR pathways

**Fig. 1:**
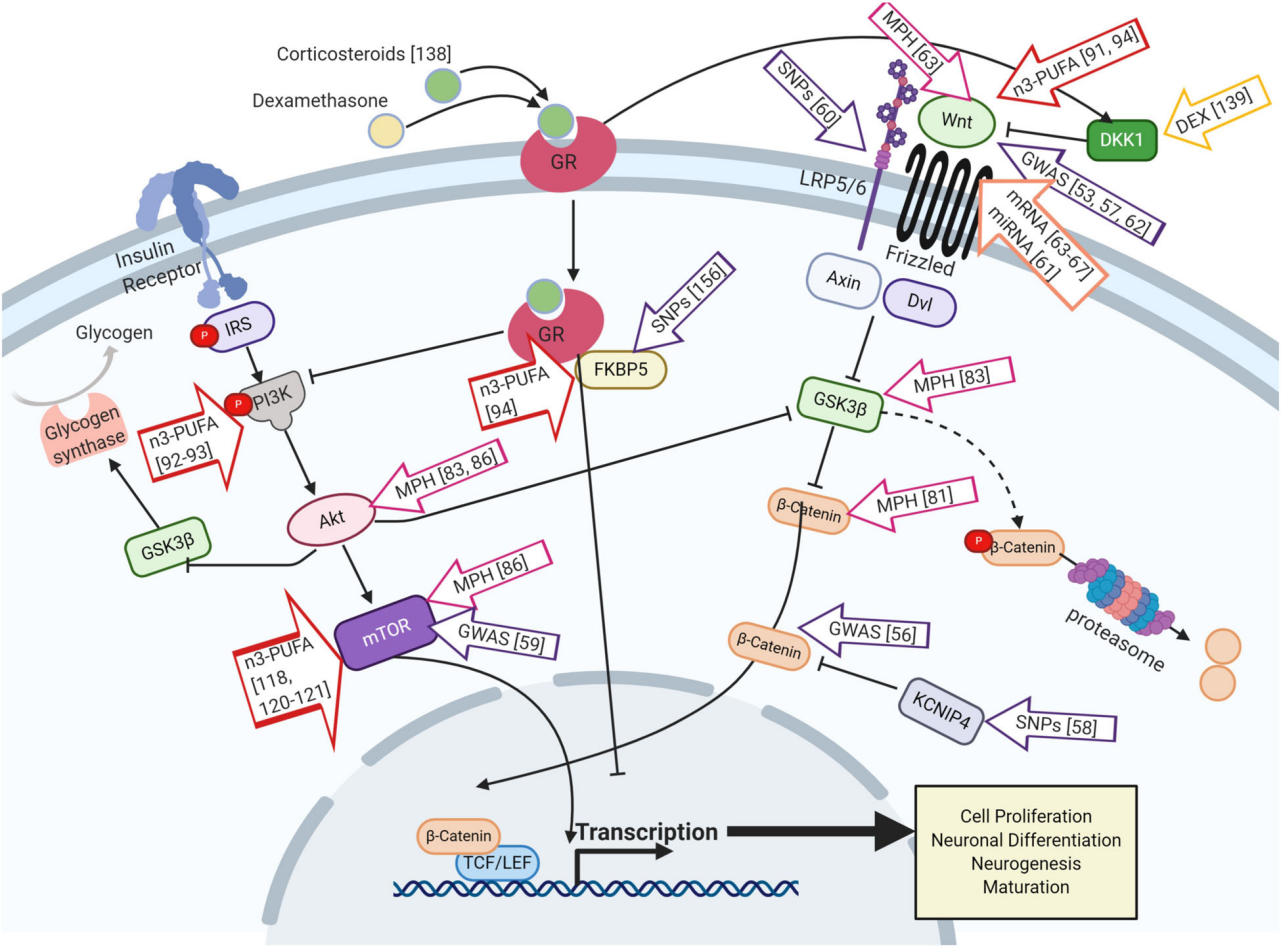
Current evidence and hypothesis linking elevated stress (cortisol), previous ADHD diagnosis, and methylphenidate (MPH) treatment or omega-3 PUFA supplementation with the Wnt and mTOR pathways known as key players in cell proliferation, growth, differentiation, and maturation. The figure was created by BioRender.com. Abbreviations: DEX dexamethasone, GR glucocorticoid receptor, GWAS genome-wide association study, miRNA non-coding microRNA, MPH methylphenidate, *n*-3-PUFA Omega-3 long-chain polyunsaturated fatty acids, SNPs single nucleotide polymorphisms, [number] represent citation in the paper.

The wingless-INT (Wnt)-signaling pathway is known to orchestrate cellular proliferation, polarity, and differentiation (Fig. 1); processes that are crucial for healthy tissue morphogenesis, especially in the embryonic stage^33^, but also processes involved in adult neurogenesis^34^. On the basis of their activity at the cellular level, Wnt-signaling has been classified into two categories, canonical and non-canonical^35^. In humans, 19 genes were identified to encode Wnt protein subtypes. Further, they activate several pathways by coupling with various receptors^36^. In the canonical Wnt-signaling pathway, Wnt ligands bind to a Frizzled (Fzd)/low-density lipoprotein receptor-related protein (LRP) complex at the cell membrane. This complex (Wnt/Fzd/LRP) recruits disheveled (Dvl), which in turn inhibits the pathway, leading to β-catenin degradation. Once the β-catenin degradation stops, it accumulates in the cytoplasm before translocating into the nucleus. Subsequently, there is an interaction between β-catenin and transcription factor (TCF)/lymphoid-enhancer factor (LEF) in the nucleus to regulate the transcription of target genes^37^. TCF/LEF target genes are known to regulate stem cell maintenance and differentiation along with cell proliferation^38^.

The mammalian target of rapamycin (mTOR) pathway is involved in several significant processes of neurodevelopment and synaptic plasticity (Fig. 1)^39^. It has also been described to be associated with neurodevelopmental disorders such as ASD^40^ and schizophrenia^41,42^. A component of the phosphatidylinositol 3-kinase (PI3K) cell survival pathway is the mTOR. It has a significant role in the regulation of cell growth and proliferation. mTOR operates at a key junction in the PI3K pathway. It further acts downstream and upstream of protein kinase Akt. mTOR also regulates the protein synthesis necessary for proliferation and cell growth by forming two multi-protein complexes, namely mTOR complex I (mTORC1) and mTOR complex II (mTORC2)^43^.

There are three main signaling pathways known to converge and regulate the mechanistic target of mTOR. First, the growth factor pathway comprised of PI3Ks, phosphoinositide-dependent kinase 1 (PDK1), and RAC-alpha serine/threonine-protein kinase (AKT1). AKT1 is activated by the phosphorylation of PDK1, which is triggered when PI3K is activated. mTOR is activated via GTP-binding protein Rheb (RHEB) when tuberous sclerosis complex (TSC2) gets phosphor-inhibited by AKT1. This results in the phosphorylation of eukaryotic translation initiation factor 4E-binding protein 1 (4E-BP1) or protein S6 kinase beta-1 (S6K1)^44^. The second pathway involves the energy-sensing arm, which responds to low or insufficient ambient cellular ATP. AMPK is phosphorylated through the serine/threonine-protein kinase STK11 (also known as liver kinase B1, LKB1). This results in TSC2-mediated mTOR inhibition. LKB1 complex activity releases inhibition of mTOR when the ATP level are abundant^45^. In the third pathway, the amino acid sensor GAP activity toward rags (GATOR1) complex (DEP domain-containing protein 5, DEPDC5; nitrogen permease regulator proteins NPRL2 and NPRL3) regulates mTOR in response to changes in levels of amino acids. In other words, GATOR1 inhibits mTOR-signaling when the level of amino acids are low. GATOR1 inhibition of mTOR is released once the amino acid levels return to normal levels^46^.

Both signaling pathways are not only important for early normal brain development but also for neurogenesis in the adult brain^47^. mTOR has been suggested to regulate neuronal differentiation and dendritic maturation^48^. Canonical Wnt-signaling has a major role in neurogenesis. During early neurogenesis, it promotes self-renewal to maintain neural progenitors. However, during mid and late neurogenesis it induces the differentiation of progenitor cells^34^.

Dysregulation of both pathways may lead to pathological brain developmental conditions. For example, Kasherman and colleagues^48^ have shown how abnormalities in the ubiquitin system, a major regulator of key signaling cascades including mTOR and Wnt, are linked to two common neurodevelopmental disorders: intellectual disability (ID) and ASD.

#### Genetic evidence for Wnt-signaling in ADHD

Although ADHD was found to be highly heritable^25,49^, no single genetic variation could be used for diagnosis or prediction of ADHD. Moreover, the consensus is that ADHD is considered polygenetic, supporting also the notion that it is not a clear-cut categorical disorder but rather the extreme end of the spectrum of a continuous behavioral trait^26^. Indeed, the polygenic risk score (PRS) analysis summarizing statistically all the SNP results from a large genome-wide association study (GWAS) has shown some promising results^50^. For example, ADHD-PRS was found to associate with symptoms of hyperactivity-impulsivity and to be mediated by white matter microstructure in brain regions that have been suggested to have a role in ADHD pathology^51,52^. In addition, ADHD-PRS was found to predict educational achievement^53^, to be linked with the severity of the clinical profile in those with high PRS^54^, and even to predict MPH response^55^. Although the etiology and pathways involved in ADHD cannot be revealed by using PRS only, this approach seems to be a powerful tool studying endophenotypes of the heterogeneous disorder.

In the recent Psychiatric Genomic Consortium (PGC)-ADHD meta-analysis of the GWAS results including over 20,000 cases and 35,000 controls, the first genome-wide significant loci were identified (12 loci)^53^. In addition to the 12 loci that covered 16 genes involved in brain-related pathways such as synapse formation, neuronal developmental, and dopaminergic transmission, gene set analysis of the most significant genes revealed that the Wnt/catenin-signaling pathway is associated with ADHD (gene ontology biological processes and cellular compartment)^53^. Further studies on gene variants of the Wnt pathway were reported in adult ADHD study using GWAS and extended pedigree linkage^56^. Here, the authors reported several genes to be associated with adult ADHD, as for example, the α-catenin (*CTNNA2*) which is known to be involved in Wnt-signaling and to have a role in the catenin–cadherin cell–adhesion complexes for synaptic transmission^56^. Similarly, Aebi and colleagues^57^, revealed a significant enrichment of the Wnt/β-catenin signaling in a subpopulation of ADHD patients with the oppositional defiant disorder (ODD) by running bioinformatics clustering of the GWAS results. In a study assessing personality disorders, adult ADHD and family-based childhood ADHD, Kv channel-interacting protein 4 (*KCNIP4*) gene, suggested to be part of a negative feedback loop in the Wnt/β-catenin pathway, was found to associate with ADHD^58^.

Interestingly, Rovira and colleagues^59^ reported recently a shared contribution of common SNPs in childhood ADHD and persistent adults in a meta-analysis of databases from large cohorts of ADHD children and persistent ADHD adults. In this study, the researchers were able to identify nine loci, containing genes involved in brain development, associated with ADHD persistence into adulthood. A significant genetic correlation between ADHD in childhood and its persistent ADHD in adulthood was found, in which also childhood ADHD-PRSs were associated with persistent ADHD^59^. Lastly, though not significant after multiple testing corrections, in the pathway analysis of persistent ADHD adults, nominal significant association to the “reactome energy-dependent regulation of mTOR by LKB1/AMPK”, “biocarta mTOR pathway”, and “reactome mTORC1-mediated signaling” was found^59^.

In a recent study by our group, we found both in family and case–control samples an association in child and adolescent ADHD with *LRP5* and *LRP6* gene variants in four Caucasian European populations, which was found to be further significant after meta-analysis with previous published results^60^. Additional evidence to the involvement of the Wnt-signaling in ADHD comes from a study by Wang and colleagues^61^ who demonstrated in ADHD patients a significant correlation between low gray matter volume and three miRNAs (miR-30e-5p, miR-126-5p, and miR-140-3p) that were found to be involved in the Wnt-signaling pathways. In a recent study, the genetic architecture of the human cerebral cortex revealed that common genetic variants are linked with surface area and thickness of these regions^62^. In particular, intriguing is that loci linked with surface area, which was found to be negatively correlated genetically with ADHD, clustered with genes involved in the Wnt-signaling pathways^62^ (Fig. 1).

Enrichment analysis of transcriptomic (gene expression/mRNA)^63^ data sets originating from mouse substantia nigra^64^, rat striatum, and frontal cortex^65^, immortalized human lymphocytes of adult ADHD and controls^66^ treated with MPH resulted in enriched transcripts in Wnt-signaling pathway. Similarly, in selective breeding for high home cage activity, the striatum transcriptomic of this mouse model for ADHD was assessed with and without amphetamine^67^. As in the previous models, also high-active mice enrichment analysis predicted the downregulation of the canonical Wnt pathway^67^.

Indeed, all the above-mentioned results support the involvement of Wnt-signaling pathways in ADHD and its phenotypes at the genetic and the transcriptomic level.

#### Methylphenidate treatment and its link to Wnt/mTOR-signaling

It is well-known that MPH is one of the first-line drugs for the treatment of ADHD but despite its relatively high effect sizes in ADHD children (0.8–1.0 for hyperactivity and 0.6–0.8 for attention deficit)^68^, its acute and chronic effects are still unclear and of continuous concern^69,70^. However, no conclusive evidence supporting or dismissing short- and long-term negative effects of MPH has been reported up to this date.

In vivo evidences showed that this stimulant affects positively hippocampal neurogenesis in young rats and neuronal plasticity in the amygdala^71^. Clinical studies using fMRI analysis report that MPH administration is able to attenuate ADHD-related impairment in cortical maturation and functionality in children and adolescents by increasing gray matter levels in their cortices^72–74^. Although in general, MPH acts as dopamine, norepinephrine, and at low-level serotonin transporter inhibitor in high-, middle and low-affinity manners, respectively, thus enhancing the levels and the biological effects of these monoamines in the synaptic cleft^75^, the exact molecular mechanisms (including the pathways affected by MPH) involved in this context remains elusive.

In vitro research has revealed that MPH influences cell proliferation and differentiation, regulating the activity of synthesizing and metabolizing enzymes responsible for balancing the monoamine concentration in the cells and the synaptic cleft^76–78^, but also acts through a novel mechanism of action that supports neuronal cell maturation^63^.

In order to test the influence of MPH on neuronal cell maturation and its capability of stimulating neurogenesis and cortical maturation in in vivo models^79^, our group has hypothesized that treatment with this drug could lead to these same effects in neural cell lines through the Wnt-signaling activation mechanism. This was hypothesized since it has a pivotal role in maintaining these cellular processes in several conditions, such as neurogenesis and differentiation in embryonic development and tissue homeostasis^33,80^. More specifically, we were able to demonstrate in neuronal cell lines (murine stem cells, rat PC12, and human SH-SY5Y cells) that MPH activates Wnt-signaling, which was not due to the dopamine transporter inhibition—the main known therapeutic mechanism of this drug—since selective dopamine transporter inhibitor GBR-12909 treatment demonstrated the opposite effects^63^. Using a Wnt-luciferase reporter assay in 3T3 mouse fibroblast cell lines, we could confirm, quantitatively, that MPH activates the Wnt-signaling^63^.

In vivo, alterations in the expression of mRNAs involved in neuronal, synaptic, and axonal processes, in differentiation and in the Wnt-signaling were observed in MPH-treated rodents^64,65^. In addition, Oakes and collaborators^81^ found that β-catenin (as well as a vascular endothelial growth factor—VEGF —and tropomyosin receptor kinase B—TrkB) levels in juvenile male mice were elevated after a low dose of chronic MPH treatment (1 mg/kg) and reduced after high dose chronic treatment (10 mg/kg) administrated by intraperitoneal injections. The initial increase in the concentration of these factors by lower doses was followed by increased proliferation and survival of hippocampal cells, indicating that they might act as mediators of these cellular processes^81^ (Fig. 1). In 2018, Bae and colleagues^82^ hypothesized that Wnt-signaling might also be involved in the pathophysiology of ASD and that genes belonging to this pathway could be possible therapeutic targets of drugs shown to be effective in the therapy of comorbidities in ASD patients, such as haloperidol and MPH. This hypothesis has arisen based on the report by Mines and colleagues^83^, who have found that an 8-day MPH administration in adult C57Bl/6J mice was able to regulate phosphorylation of GSK3 and Akt, which suggests the participation of the drug in this pathway (Fig. 1). Nevertheless, both drugs have a distinct mechanism of action, as haloperidol is mainly a dopamine D2 receptor antagonist used for the treatment of irritability and aggression in ASD, while MPH is a dopamine transporter inhibitor used to treat comorbidity of ADHD in ASD patients.

The concomitant pathway to Wnt, mTOR, was also implicated in ADHD pathophysiology given that the complex Akt/mTOR-signaling is implicated in cell survival, synaptic plasticity, and formation, neurogenesis, and memory consolidation^84^. Several mutations in participating proteins on this pathway—for example, TSC1/2, RHEB, phosphatase tensin homolog (PTEN), and neurofibromin (NF1)—were described to be implicated with neurological disorders such as ASD, epilepsy, and ADHD^85^.

Schmitz and colleagues^86^ reported that MPH alters the mTOR pathway at different levels by decreasing the activity of Akt and mTOR substrates after short term treatment and increasing them after long-term treatment, which might regulate the aforementioned neuronal processes according to time of treatment (Fig. 1). Based on the aforementioned data, such evidence supports the notion of participation of Wnt- and Akt/mTOR-signaling in ADHD pathophysiology and reinforces the hypothesis that MPH might directly affect these two key pathways. In this sense, more studies are required to identify the molecular mechanisms involved at the cellular level in order to clarify and elucidate possible therapeutic targets.

#### Omega-3 fatty acids, ADHD, and Wnt/mTOR-signaling

Omega-3 long-chain polyunsaturated fatty acids (*n*-3-PUFA) are essential fatty acids for homeostasis acquired through diet and include eicosapentaenoic acid (EPA), alpha-linolenic acid (ALA), and docosahexaenoic acid (DHA), which are crucial for brain development^87,88^.

At the molecular level, it is known that *n*-3-PUFA has crucial roles in metabolic processes and specific functions in neural cells such as neurogenesis, neuroinflammation, and neurotransmission^89^. In this context, DHA was able to activate Wnt-signaling in neural cells, promoting upregulation of endogenous Wnt-regulated genes including PROX1 (an adult neurogenesis marker) and increased cell survival as well as neurite outgrowth in neurons^90^. Similarly, EPA, as well as DHA treatment, promoted differentiation of induced pluripotent stem cells (iPSC)-derived astrocytes from MDD patients concomitantly with increased CREB activity^91^, also known to be regulated by the non-canonical Wnt-signaling.

These compounds were described in a diverse neurological context such as neuroinflammation. In LPS (lipopolysaccharides)-induced neuro-injured mice and PC12 cells, *n*-3-PUFA treatment increased cellular proliferation and migration while inhibiting apoptosis^92^. In addition, Shi and colleagues^92^ have reported that these events were associated with the PI3K-signaling upregulation, which subsequently led to Akt activation and to an increased expression of β-catenin. These results point to a possible simultaneous activation and connection between mTOR- and Wnt-signaling pathways (Fig. 1).

Another study has also demonstrated that the PI3K/Akt and mTORC2 pathways were directly related to neuroprotective effects of *n*-3-PUFA in vitro. In this study by Descorbeth and colleagues^93^, DHA treatment (1–200 µM) protected Schwann cells from lipotoxicity-mediated cell death through the activation of the aforementioned pathways (Fig. 1).

In a whole transcriptomic study of brain hemispheres of C57BL/6J male mice fed with either standard lab diet (SD) or with a customized fish oil-enriched diet (FD), genes involved in cellular growth and proliferation, cellular development, embryonic development, synaptic transmission, development of the brain, inflammatory response, and developmental disorders have been found to differential express following FD^94^. Interestingly, the FK506 binding protein 5 (*FKBP5*) gene, known to be involved in stress regulation (see details under in chapter Stress and ADHD), was upregulated following FD pointing to the beneficial effects of fish oil supplementation^94^ (Fig. 1). Moreover, a vast number of genes in the Wnt-signaling pathway were differentially regulated following FD supplementation^94^.

In addition, the nonpharmacological protective mechanisms of *n*-3-PUFA have been largely explored in several neurological diseases^95–99^. Evidence for alterations in *n*-3-PUFA blood levels in ADHD as well as lack of prenatal *n*-3-PUFA intake associated with the risk of ADHD has been reported. In addition, several clinical trials hint at the improvement of ADHD core symptoms after add-on *n*-3-PUFA treatment. Researchers have recently shown that children with ADHD tend to eat less Omega-3-enriched food (such as fish and seafood) in comparison to neurotypical individuals^100–102^. Indeed, according to a meta-analysis, child and adolescent ADHD patients have lower levels of DHA, EPA, and total *n*-3-PUFAs compared to controls^103^. However, the link between levels of *n*-3-PUFA and the occurrence of this disorder in childhood is not limited to postnatal exposure: Lopez-Vicente and colleagues^104^ have reported that an increase in the ratio between *n*-6 and *n*-3 concentrations in cord plasma during pregnancy may increase ADHD rates when children reach the age of seven. Some investigations have demonstrated that cognitive functions, such as working memory, teacher-rated behavior, and impulsivity, have improved after *n*-3-PUFA administration in children with ADHD^105–109^. In particular, EPA supplementation seems to elicit small but significant improvement in inattention and hyperactivity symptoms in ADHD patients^103,110,111^. An in vitro study has recently shown that PC12 dopaminergic cells had their viability significantly increased after *n*-3-PUFA administration^112^, starting with lower doses (from 100 pM). Interestingly, a double-blinded report revealed that a combined treatment of *n*-3-PUFA plus MPH in a group of ADHD children was able to increase the efficacy of MPH treatment by significantly increasing the response to this drug^113^. Similarly, Anand and colleagues^114^ found that *n*-3-PUFA could improve ADHD scores in atomoxetine-treated children in comparison to the group that received this drug only. However, the difference was not statistically significant in cases of inadequate dosage administration^114^.

In addition, Liu and colleagues^97^ have described that PUFA may modulate different signaling pathways in the brain by increasing the activity of adenylate cyclase and protein kinase A and thereby influencing serotonin, beta-adrenergic, and dopamine receptors.

Although, to date, no link was established between *n*-3-PUFA, mTOR, and ADHD some evidence has been reported for *n*-3-PUFA and mTOR in other neuropsychiatric disorders. Based on mounting evidence proving that *n-*3-PUFA have beneficial effects on people affected by depression, schizophrenia, and bipolar disorder^115–117^ in combination with data on the correlation between mTOR and other pathologies (e.g., cancer), Shirooie and colleagues^118^ reviewed that mTOR can be one of *n*-3-PUFA’s targets to treat neurodegenerative diseases by regulating essential cellular processes such as apoptosis and autophagy. In addition, Giacobbe and colleagues^119^ have recently discussed the anti-inflammatory effect of *n*-3-PUFA within the context of neuropsychiatric disorders such as depression. This review mentions that intracerebroventricular injections of resolvins D1, D2, and E1 (*n*-3-PUFA metabolites) seem to attenuate depressive-like behavior in mice via activation of mTORC1-signaling pathway^120,121^. Therefore, *n*-3-PUFA might elicit its positive effects on ADHD via mTOR, however, this still needs to be investigated.

To date, there is a lack of evidence connecting Wnt and/or mTOR pathways, *n*-3-PUFA supplementation, and the occurrence of ADHD. Moreover, some results are controversial to the aforementioned positive studies about favorable aspects of *n*-3-PUFA supplementation^122,123^. Although overall results point to its beneficial effects mainly in mild ADHD subtypes^124^, making deeper and molecular approaches even more necessary.

#### Stress and ADHD

As mentioned previously, the susceptibility to ADHD is influenced by the interaction of polygenic and environmental factors. The impact of environmental risk factors increases especially during critical developmental stages in life. The pre- and perinatal periods are associated with high phenotypic plasticity and thereby represent such critical developmental episodes^125,126^. Risk factors such as maternal life-events such as stress or trauma, alcohol consumption, and smoking during pregnancy put offspring at risk of developing ADHD^127–129^. However, the degree to which these are casual effects or due to unknown confounding factors is highly debated^130,131^. Recent studies suggested that risk factors, such as maternal substance use during pregnancy is associated with offspring ADHD, are not casual but rather confounding by other risk factors^132,133^.

In terms of maternal influence on ADHD development, maternal ADHD symptomatology, also in mothers without ADHD diagnosis, may indirectly induce ADHD symptomatology in the offspring by negatively affecting the home environment^134^. Moreover, the presence of depressive symptoms during pregnancy suggested inducing the early onset of ADHD symptoms in 3- to 6-year-old children^135^. Therefore, it is rather difficult to separate pre, peri-, and postnatal stress effects on the risk of ADHD, however the overall hypothesis of accumulating environmental factors including stress may exacerbate it.

In addition, socioeconomic factors seem to be an essential role in this context, since children whose mothers are from urban, low-income backgrounds and had been under psychological stress before and during pregnancy are more susceptible to develop ADHD^136^. Such stress may result directly from maternal cortisol levels, indirectly from maternal anxiety, depression, trauma, or even from the administration of synthetic glucocorticoids such as dexamethasone or betamethasone^137^. Glucocorticoids can influence intracellular signaling pathways as for example the Wnt-signaling pathways (Fig. 1). An increase in glucocorticoids has been shown to downregulate both ERK- and PI3K/Akt-signaling pathways^138^. Treatment of human neural progenitor cells (hNPCs) with dexamethasone for example increases the expression of Dickkopf 1 (DKK1) and decreases the expression of canonical Wnt target genes^139^. To note, the Wnt-signaling is important for neurogenesis and its inhibition causes a defect in neural differentiation and proliferation, which might explain how stress affects neurodevelopment^80,139^. The neuronal and glial energetics hypothesis further describes a possible association between glucocorticoids and psychiatric neurodevelopmental disorders. Glucocorticoids inhibit glycogen synthesis as well as the supply of glucose to neurons and astrocytes^140^. As a result, the glial activity will be downregulated during stressful situations and will lead to fatigue and a predisposition for an inefficient and/or inconsistent behavioral performance^140^.

It has been confirmed in animal models that ADHD-like behavioral symptoms and changes in brain function can result from early life stress^141,142^. The spontaneous hypertensive rat (SHR) is one of the most extensively studied animal models for ADHD^143^. Cognitive performance tests in SHR indicate an association between cognitive deficits, insulin resistance, and hypertension^144^, while Wnt-signaling has been shown to mediate insulin and blood pressure regulation and thereby suggests a possible relationship between Wnt-signaling and ADHD^145^.

ADHD symptoms are associated with higher perceived stress^146,147^. The hypothalamus–pituitary–adrenal axis (HPA axis) is an important responder to stressful environments. Several studies have shown that stress reactions are different in ADHD patients compared to healthy controls^148–151^. ADHD patients show reduced diurnal cortisol levels compared to healthy controls^152,153^. Cortisol levels can be decreased by mineralocorticoids- (MR) and glucocorticoids- (GR) receptors through a negative feedback system. GR and MR inhibit the synthesis and release of corticotropin-releasing factor in the hypothalamus and adrenocorticotrophic hormone in the pituitary gland^154^. The candidate gene *FKBP5* is involved with the GR and its pathway^155,156^. It is an important regulator of the negative feedback system of the HPA axis by reducing the sensitivity of GR^157^. Stress hormones increase the expression of the FKBP5 resulting in reduced GR affinity and thereby increase cortisol levels. Polymorphisms on the *FKBP5* gene have been found to be associated with ADHD^156^. Moreover, one of the polymorphisms was found to associate with low cortisol levels and ADHD. Therefore, FKBP5 might have an important role in linking Wnt-signaling, stress, and ADHD (Fig. 1).

Preclinical studies^158–160^ suggest that *n*-3-PUFAs improve stress-related neurobiological and behavioral changes via attenuation of the associated HPA-axis hyperactivity. The latter findings are suggestive that dietary *n*-3-PUFAs may modulate the often observed HPA-axis dysfunction associated with the development of some neuropsychiatric disorders including ADHD and MDD. Observational studies in humans further support a link between the HPA axis and PUFA metabolism, as cortisol decreases the production^161^ and increases the breakdown of PUFAs^162^.

## Discussion

From the current review of the literature, we suggest the involvement of Wnt and mTOR pathways in the etiopathology of ADHD with a wide range of evidence, at the genetic, environmental, functional, and molecular as well as at the pharmacotherapeutic levels. Nevertheless, there is still a gap between the current knowledge and the lack of evidence in neuronal brain tissue originating from ADHD patients. This, of course, arises because brain tissue is not available since neither biopsies nor post-mortem samples are available.

Based on the data presented so far, it is noticeable that genetic factors are relevant in understanding ADHD. Studying such genetic risk factors at the molecular level is as important as exploring the environmental factors that might have a role in the disorder. To illustrate this, we pointed out the possible involvement of pre-, peri-, and postnatal stress associated with ADHD also linking it to the genetic risk factors, possibly together affecting canonical Wnt-signaling, which in turn affects neurodevelopment. Unfortunately, there is still a shortcoming in understanding the role of stress on the molecular and cellular levels that needs to be elucidated.

Studying the pathomechanism in human cells is limited because the relevant tissue, the brain, is not accessible by biopsy and ADHD post-mortem samples are scarcely available for research purposes. Although animal models are a valuable alternative in studying ADHD, they are not able to mimic the polygenic profile of a patient^163^. Therefore, there is an urgent need for a personalized neuronal model in a dish originating from individuals with ADHD that maintains their genetic background. Reprogramming human somatic cells into iPSCs and differentiating them into neural cells or brain organoids is a powerful technology that provides the essential means to study living human central nervous system cells for modeling complex disorders such as ADHD in a semi ex vivo manner^164^. Such personalized models may provide a platform to test such a hypothesis, enabling the measures of molecular and functional effects of genetic and environmental factors on neurodevelopment processes.

In conclusion, both pathways may be associated with ADHD at the genetic and environmental level linking both susceptible risk genes with stress. Indeed, Wnt-/mTOR-signaling and stress were linked with many other neurodevelopmental and neurodegenerative disorders as reviewed above. This does not contradict to the fact that many of these disorders are comorbid as well as share some phenotypes, e.g., cognition, which might explain the common mechanisms. However, the polygenic combination, the time point of alterations, and the time point of stress might cause to the different trajectories leading to the disorder, as reviewed by Sullivan and colleagues^165^. Following the aforementioned observations, it would be of utmost importance to test the hypothesis of Wnt- and mTOR-pathway alterations and stress effects at the cellular level in developing neural cells from ADHD patients compared to healthy controls. As discussed previously, the novel technique of using iPSCs to establish a personalized model of ADHD seems to be a novel platform to deeply understand the mechanism of ADHD, which will also provide scope for drug testing. Overall, it would be an important step forward to form not only effective treatment for ADHD but also for the development of preventive approaches.
